# Green tea extract catechin improves cardiac function in pediatric cardiomyopathy patients with diastolic dysfunction

**DOI:** 10.1186/s12929-019-0528-7

**Published:** 2019-05-08

**Authors:** Junjun Quan, Zhongli Jia, Tiewei Lv, Lei Zhang, Lingjuan Liu, Bo Pan, Jing Zhu, Ira J. Gelb, Xupei Huang, Jie Tian

**Affiliations:** 1 Department of Cardiology, Children’s Hospital of Chongqing Medical University, 136 Zhongshan Er Road, Yu Zhong District, Chongqing, 400014 China; 2 Ministry of Education Key Laboratory of Child Development and Disorders, Chongqing, China; 3 China International Science and Technology Cooperation Base of Child Development and Critical Disorders, Chongqing, China; 4 Chongqing Key Laboratory of Pediatrics, Chongqing, China; 5 Charlie E. Schmidt College of Medicine, Florida Atlantic University, 777 Glades Road, Boca Raton, FL 33431 USA

**Keywords:** Hypertrophic cardiomyopathy, Restrictive cardiomyopathy, Diastolic dysfunction, Green tea extract catechin

## Abstract

**Background:**

Our previous studies have demonstrated that Ca^2+^ desensitizing catechin could correct diastolic dysfunction in experimental animals with restrictive cardiomyopathy. In this study, it is aimed to assess the effects of green tea extract catechin on cardiac function and other clinical features in pediatric patients with cardiomyopathies.

**Methods:**

Twelve pediatric cardiomyopathy patients with diastolic dysfunction were enrolled for the study. Echocardiography, ECG, and laboratory tests were performed before and after the catechin administration for 12 months. Comparison has been made in these patients before and after the treatment with catechin. Next Generation Sequencing was conducted to find out the potential causative gene variants in all patients.

**Results:**

A significant decrease of isovolumetric relaxation time (115 ± 46 vs 100 ± 42 ms, *P* = 0.047 at 6 months; 115 ± 46 vs 94 ± 30 ms, *P* = 0.033 at 12 months), an increase of left ventricle end diastolic volume (40 ± 28 vs 53 ± 28 ml, *P* = 0.028 at 6 months; 40 ± 28 vs 48 ± 33 ml, *P* = 0.011 at 12 months) and stroke volume (25 ± 16 vs 32 ± 17 ml, *P* = 0.022 at 6 months; 25 ± 16 vs 30 ± 17 ml, *P* = 0.021 at 12 months) were observed with echocardiography in these patients 6-month after the treatment with catechin. Ejection fraction, left ventricular wall thickness, biatrial dimension remained unchanged. No significant side effects were observed in the patients tested.

**Conclusions:**

This study indicates that Ca^2+^ desensitizing green tea extract catechin, is helpful in correcting the impaired relaxation in pediatric cardiomyopathy patients with diastolic dysfunction.

**Electronic supplementary material:**

The online version of this article (10.1186/s12929-019-0528-7) contains supplementary material, which is available to authorized users.

## Introduction

Cardiomyopathy is a common heart disease in children that leads to cardiac dysfunction. Among three major types of cardiomyopathies, hypertrophic cardiomyopathy (HCM), dilated cardiomyopathy (DCM) and restrictive cardiomyopathy (RCM), HCM and RCM share a common pathological feature, i.e. diastolic dysfunction whereas the main manifestation in DCM is systolic dysfunction [1]. In addition, HCM is most likely responsible for sudden cardiac death in the young [2, 3]. Although RCM is the least common, accounting for approximately 5% of the total pediatric cardiomyopathies, it has a very poor prognosis with about 50% deaths 2 years after diagnosis [4]. Except for cardiac transplantation, there have been no effective treatments for cardiomyopathy patients with diastolic dysfunction since its mechanisms are still unknown [5–7].

Recently, several laboratories including ours have demonstrated that cardiac myofibril hypersensitivity to Ca^2+^ is one of key factors resulting in an impaired relaxation in myocardial cells [8]. Using transgenic mouse model of cardiomyopathy, we have for the first time elucidated the relationship between Ca^2+^ hypersensitivity caused by myofibril protein mutations and the following diastolic dysfunction in the heart [8]. Furthermore, we have confirmed that desensitization, either using a transgenic molecule [9] or green tea extract catechin [8], is a useful tool to correct diastolic dysfunction caused by Ca^2+^ hypersensitivity in cardiomyopathy. Besides, the same process has been observed in HCM mouse model [10]. Experiments in vivo have indicated that calcium desensitizing catechin can interact with cardiac troponin-C to reduce myofilament Ca^2+^ hypersensitivity [11]. Due to its properties, catechin has been used to improve impaired heart relaxation and diastolic dysfunction in HCM or RCM [8, 10, 11]. In the present study, we have tried to confirm the therapeutic effects of green tea extract catechin on diastolic dysfunction in pediatric cardiomyopathy patients by comparing the cardiac function before and after the treatment with catechin.

## Materials and methods

### Study subjects

Fourteen patients were admitted to Children’s Hospital of Chongqing Medical University (Chongqing, China) from October 2015 to January 2017 due to HCM or RCM. Eligible patients were ≤ 18 years of age, were diagnosis as HCM or RCM, and had evidence of diastolic dysfunction confirmed by echocardiography and clinical manifestations. Diagnostic criteria for HCM are: left ventricular hypertrophy with wall thickness ≥ 2 standard deviations above the mean (z score ≥ 2) for age, sex, or body size, and absence of defined metabolic or hemodynamic causes such as metabolic disorders, congenital heart disease, hypertension, or exposure to drugs known to cause cardiac hypertrophy [12–14]. Diagnostic criteria for RCM are: dilated atria, evidence of diastolic dysfunction with normal or nearly normal left ventricular systolic function, no evidence of significant left ventricular hypertrophy or dilation, and absence of congenital, valvular, or pericardial diseases [15, 16]. Finally, two patients were excluded due to the diagnosis of Pompe disease by genotyping and protein analysis and a total of 12 patients were enrolled in this study. Clinical data including medical history (age at admission, gender, weight, height, family history and clinical symptoms), physical signs, results of the diagnostic examinations (laboratory tests, ECG, echocardiography, chest X-ray, abdominal ultrasonography and cardiac magnetic resonance imaging) and treatments were collected and recorded. Written informed consent was obtained from legal guardians of all participating patients.

All participating patients were orally administrated with green tea extract catechin on their own or their legal guardian’s initiative. Green tea *(Camillia sinensis)* contains health-promoting polyphenol compounds like epigallocatechin-3-gallate (EGCG). Decaffeinated Mega Green Tea Extract is manufactured by Life Extension Inc. (Life Extension Inc., Fort Lauderdale, FL, USA) and it contains more of these potent compounds than several cups of green tea in a convenient, once-daily, decaffeinated supplement (325 mg EGCG/capsule). The initial dose of catechin was one capsule/day (about 15 mg/kg daily) and added up to three capsules/day (about 50 mg/kg daily) in 3 months after catechin initial administration [8]. The half-life of EGCG is about 13–14 h [17]. All patients were administrated with green tea catechin for 12 months. In addition, all patients received routine treatment (for example, diuretics, beta-blockers, calcium channel blocking drugs, beta-adrenergic blocking agents, and angiotensin-converting enzyme inhibitors (ACEI), etc.) in accordance with the guideline for the treatment of HCM or RCM [13, 18]. Follow-up investigation was carried out by self-comparison between before and after the catechin administration. Echocardiography, laboratory tests and ECG were performed before and after the treatment of catechin.

### Echocardiography

Transthoracic echocardiography measurements were performed with an ultrasound diagnostic system (IE33, Philips Inc., Amsterdam, Netherlands) by two experienced sonographers who were blinded about all other clinical data and were conducted according to the recommendations by American Society of Echocardiography. Heart rate and blood pressure were measured and recorded while the echocardiography was performed. Systolic and diastolic functions were obtained from M-mode and pulse-wave Doppler imaging. Under short-axis view, thicknesses of interventricular septum (IVS) and left ventricular (LV) posterior wall were determined in late diastole using M-mode imaging, and under four-chamber view, bi-atrial size was scaled as well. Pulmonary arterial pressure (PAP) was estimated by measuring right ventricle systolic pressure (RVSP) using tricuspid regurgitation velocity. The cardiac diastolic function and mitral blood inflow were detected using the pulse-wave Doppler. In addition, the E/A ratio (E wave, early ventricular filling; A wave, late ventricular filling) and isovolumetric relaxation time (IVRT) were measured as well. Left ventricular diastolic dysfunction was defined as IVRT < 40 or > 80 ms or mitral E/A < 1 or > 2. Data analysis was carried out off-line using a customized version of self-contained analytic software from ultrasonic apparatus.

### Laboratory tests

Peripheral total blood was collected using tubes containing inertia separation gel and coagulants without shaking for laboratory analyses, including high-sensitivity troponin I (hsTnI), myoglobin (MYO), creatine kinase isoenzyme (CKMB) and brain natriuretic peptide (BNP), and alanine aminotransferase (ALT), aspertate aminotransferase (AST), lactate dehydrogenase (LDH) and gamma-glutamyl transpeptidase (GGT) of hepatic function, and blood urea nitrogen (BUN), creatinine (CREA), uric acid (URCA) of renal function. All tests were conducted by Clinical Laboratory Center of Children’s Hospital of Chongqing Medical University (Chongqing, China).

### Genetic tests

Peripheral blood samples from all patients and their family were collected using tubes containing ethylene diamine tetra-acetate for Next Generation Sequencing (Beijing, JinZhun, Gene, Technology, Co, Ltd., China) and Sanger Validation, respectively. Subsequently, the pedigree of pathogenic or likely pathogenic mutations was processed to analyze genotypes and phenotypes of the diseases.

### Statistical analysis

Categorical variables were presented as absolute number and percent. Continuous data were presented as mean ± SD. All data analyses were performed with the use of SPSS (version 22.0, IBM Corporation, Armonk, New York, USA). Differences were compared by the method of Wilcoxon matched-pairs signed-rank test, which between nonparametric, continuous data detected at study inclusion and at 6-month, and at study inclusion and 12-month follow-up. Statistical significance was considered when *P*-value was less than 0.05.

## Results

### Patients and characteristics

Twelve pediatric cardiomyopathy patients with diastolic dysfunction were included (five HCM and seven RCM, age ranges from 0.8 to 14.2 years). During the study, three patients died of sudden death and heart failure were terminated from the study. The remaining patients continued the whole study and no case withdrawal of the catechin administration during the whole study period. No significant complains or side effects were recorded or observed in these patients after administration of catechin. All patients but two have an obvious family history. The main clinical data and general conditions of all enrolled patients are listed in Table 1.

**Table 1:** General characteristics and manifestation of the tested patients

	All patients
Age (years)	6.8 ± 5.1
Gender: male/female	8/4
Body weight (kg)	21.4 ± 11.4
Height (cm)	113.2 ± 33.7
BMI (kg/m^2^)	16.1 ± 3.1
Diagnosis	
HCM	5
RCM	7
Family history	2
Symptoms	
Exercise intolerance	12
Syncope	3
Signs	
Cardiomegaly	12
Hepatomegaly	9
Edema	6
Ascites	3
Patients with gene mutation	9

Notes: *BMI* body mass index, *HCM* hypertrophic cardiomyopathy, *RCM* restrictive cardiomyopathy. Continuous data are presented as mean ± SD and categorical variables are presented as number (percent)

According to New York Heart Association (NYHA) heart failure classification criteria (Additional file 1: Table S6) [19], the remaining nine subjects all showed a significant improvement in cardiac function after the administration of catechin (heart failure levels in these patients were changed from Class III to II or from Class IV to III after the administration of catechin). The follow-up time was arranged from 4 to 27 months (mean: 16 months) after the treatment. Cardiac function changes based on NYHA classification in the subjects before and after the catechin administration are shown in Table 2. No significant alterations in 12-leads ECG (heart rate, P, P-R, QRS, QT, QTc) were observed in all patients receiving the daily catechin administration (Additional file 1: Table S1).

**Table 2:** Genetic analysis, changes of NYHA class and prognosis before and after the catechin administration

NO.	Diagnosis	Gene (site)	Amino acid (clinical significance)	Carrier	NYHA class		Follow-up (months)	Prognosis
					Pre-catechin	Post-catechin		
1	HCM	MYH7 (761C > A)	Ala254Glu (VUS)	None^b^	III	II	7	Died (SD)
2	HCM	MYH7 (2464A > G)	Met822Val (Pathogenic)	None	III	II	27	Alive
3	HCM	MYH6 (3640C > T) RAF1 (775 T > A)	Arg1214Trp (VUS) Ser259Thr (Pathogenic)	Mother None	III	II	24	Alive
4	HCM	TPM1 (900-4G > A) RAF1 (770C > T)	Splicing (VUS)Ser257Leu (Pathogenic)	Mother None	IV	IV	4	Died (HF)
5	HCM	NEXN (835C > T)	Arg279Cys (VUS)	Father	III	II	12	Alive
6	RCM	TNNI3 (574C > T)	Arg192Cys (Pathogenic)	None	IV	II	24	Alive
7	RCM	PKP2 (2246C > A) TNNI3 (575G > A)	Ala749Asp (Pathogenic) Arg192His (Pathogenic)	None None	III	II	16	Alive
8	RCM	DSP (4943A > G) DSP (6223C > T) ILK (707A > G)	Gln1648Arg (VUS) Arg2075Trp (VUS) Asn236Ser (VUS)	Father Mother Mother	IV	IV	4	Died (HF)
9	RCM	MYH7 (3854-5C > T)	Splicing (VUS)	None	III	II	21	Alive
10	RCM	Undetected^a^	Undetected	/	IV	III	27	Alive
11	RCM	Undetected	Undetected	/	III	II	14	Alive
12	RCM	Undetected	Undetected	/	III	II	13	Alive

Notes: *DSP* desmoplakin, *HCM* hypertrophic cardiomyopathy, *HF* heart failure, *ILK* integrin linked kinase, *MYH6* alpha-myosin heavy chain, *MYH7* beta-myosin heavy chain, *NYHA* New York Heart Association, *NEXN* nexilin F-actin binding protein, *PKP2* plakophilin, *RAF1* Raf-1 proto-oncogene, serine/threonine kinase, *RCM* restrictive cardiomyopathy, *SD* sudden death, *TNNI3* isoform of troponin I, *TPM1* tropomyosin alpha-1 chain, *VUS* variants of uncertain significance. ^a^ There are no variants detected in patients. ^b^ There are no carriers found in patients’ family

### Echocardiography analysis

Mean IVS thickness and LV posterior wall thickness in HCM patients, and mean dimension of left and right atria in RCM patients remained unchanged after the catechin administration (Additional file 1: Table S2–3 and Additional file 2: Figure S1).

**Fig. 1:**
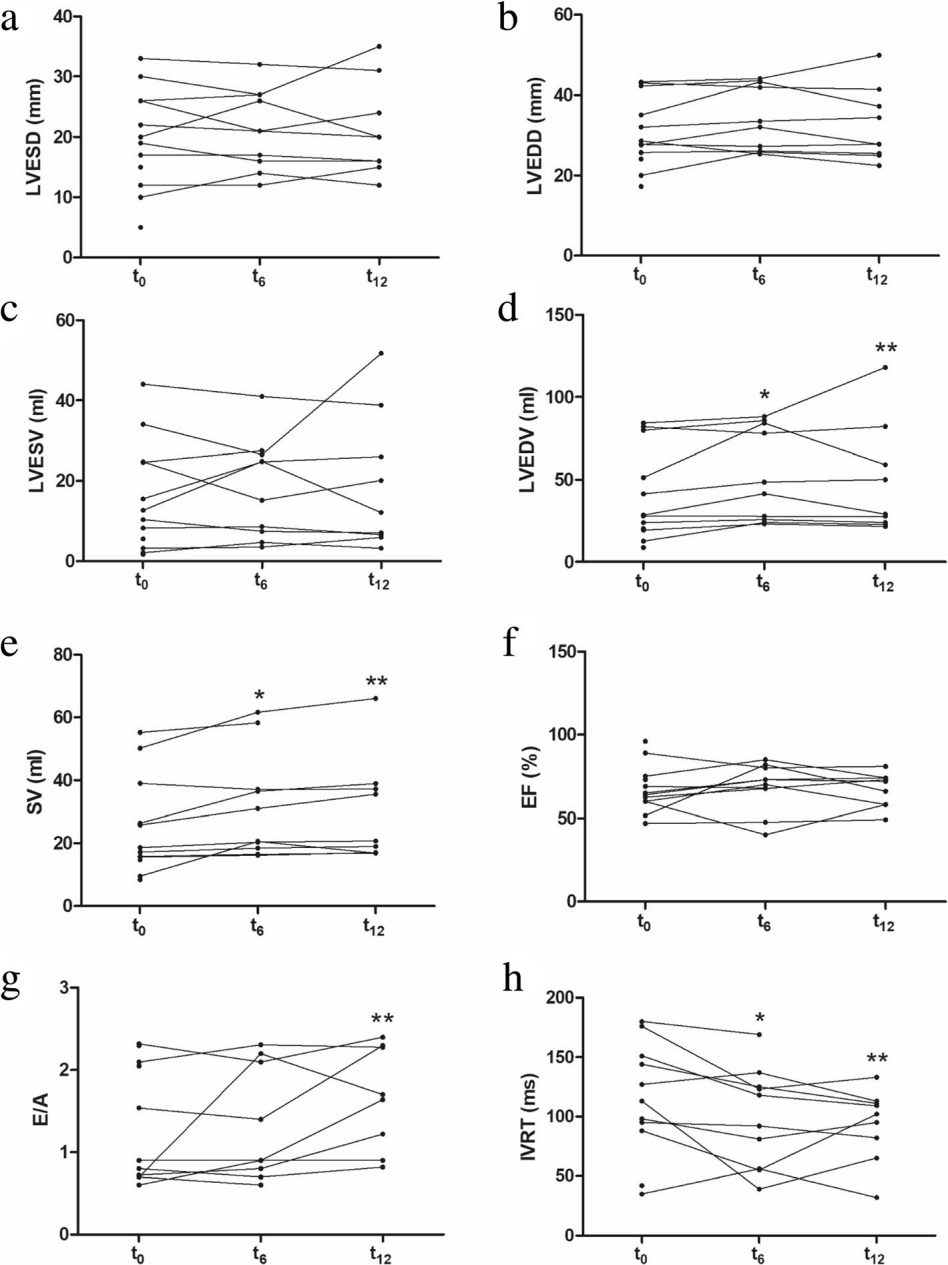
Effects of Ca^2+^ desensitizing green tea extract catechin on cardiac function in cardiomyopathy patients with diastolic dysfunction. Various cardiac functions have been measured using echocardiography in cardiomyopathy patients before (t_0_) and after (t_6_ or t_12_) catechin treatment: (**a**) left ventricular end systolic dimension (LVESD); (**b**) left ventricular end diastolic dimension (LVEDD); (**c**) left ventricular end systolic volume (LVESV); (**d**) left ventricular end diastolic volume (LVEDV); (**e**) stroke volume (SV); (**f**) ejection fraction (EF); (**g**) mitral Doppler E/A wave ratio (E/A ratio); (**h**) isovolumetric relaxation time (IVRT). t_0_, before the administration of catechin; t_6_, 6 months after the administration of catechin; t_12_, 12 months after administration of catechin. ^*^ *P* < 0.05, t_6_ vs t_0_; ^**^ *P* < 0.05, t_12_ vs t_0_

Left ventricle end systolic dimension (LVESD, 20 ± 8 vs 21 ± 7 mm, *P* = 0.778 at 6 months after catechin treatment; 20 ± 8 vs 21 ± 8 mm, *P* = 1 at 12 months after catechin treatment), left ventricle end diastolic dimension (LVEDD, 31 ± 9 vs 34 ± 8 mm, *P* = 0.139 at 6 months after catechin treatment; 31 ± 9 vs 32 ± 9 mm, *P* = 0.343 at 12 months after catechin treatment), and left ventricle end systolic volume (LVESV, 16 ± 14 vs 18 ± 12 mm, *P* = 0.959 at 6 months after catechin treatment; 16 ± 14 vs 19 ± 17 mm; *P* = 0.953 at 12 months after catechin treatment) remained unchanged after catechin administration. Left ventricle end diastolic volume (LVEDV, 40 ± 28 vs 53 ± 28 ml, *P* = 0.028 at 6 months; 40 ± 28 vs 48 ± 33 ml, *P* = 0.011 at 12 months) was increased by 17.1% in most patients after catechin administration for 6 months and by 17.0% after catechin administration for 12 months. A significant improvement of stroke volume (SV) by 14.9% in most patients was observed after 6-month catechin administration, and by 23.0% in all patients after 12-month catechin treatment. However, ejection fraction (EF) and fractional shortening (FS) of LV remained stable before and after catechin administration. The E/A ratio (1.3 ± 0.7 vs 1.3 ± 0.7, *P* = 0.674) remained unchanged in the patients after 6-month catechin treatment. An improvement of the E/A ratio (1.3 ± 0.7 vs 1.7 ± 0.6, *P* = 0.018) was observed in the patients after 12-month catechin treatment. A significant decrease of IVRT was observed after catechin administration for 6 months or 12 months. PAP calculated by RVSP remained unchanged, along with three patients having pulmonary arterial hypertension. Heart rate and blood pressure remained changeless in the patients tested during the study. In addition, five patients with a longer term of follow up (21–27 months) showed a significant reduction of IVRT in echocardiography analysis. Cardiac functions measured with echocardiography are exhibited in Table 3 and Fig. 1.

**Table 3:** Cardiac function measured with echocardiography

Parameter	Study inclusion (*n* = 12)	6 months after study start (*n* = 10)	*P*-value^*^	12 months after study start (*n* = 9)	*P*-value^**^
HR (bpm)	93 ± 26	91 ± 18	ns	86 ± 19	ns
Systolic BP (mmHg)	96 ± 18	104 ± 12	ns	97 ± 16	ns
Diastolic BP (mmHg)	59 ± 12	60 ± 5	ns	57 ± 15	ns
LVESD (mm)	20 ± 8	21 ± 7	ns	21 ± 8	ns
LVEDD (mm)	31 ± 9	34 ± 8	ns	32 ± 9	ns
LVESV (ml)	16 ± 14	18 ± 12	ns	19 ± 17	ns
LVEDV (ml)	40 ± 28	53 ± 28	0.028	48 ± 33	0.011
SV (ml)	25 ± 16	32 ± 17	0.022	30 ± 17	0.021
EF (%)	68 ± 14	69 ± 14	ns	67 ± 10	ns
FS (%)	38 ± 13	39 ± 11	ns	37 ± 7	ns
E/A	1.3 ± 0.7	1.3 ± 0.7	ns	1.7 ± 0.6	0.018
IVRT (ms)	115 ± 46	100 ± 42	0.047	94 ± 30	0.033
RVSP (mmHg)	29 ± 8	30 ± 6	ns	30 ± 5	ns

Notes: *A* mitral Doppler A peak velocity, *BP* blood pressure, *E* mitral Doppler E peak velocity, *EF* ejection fraction, *FS* fractional shortening of LV, *HR* heart rate, *IVRT* isovolumetric relaxation time, *LVEDD* left ventricle end diastolic dimension, *LVEDV* left ventricle end diastolic volume, *LVESD*, left ventricle end systolic dimension, *LVESV* left ventricle end systolic volume, *ns* nonsignificance, *RVSP* right ventricle systolic pressure, *SV* stroke volume. Continuous data are expressed as mean ± SD. ^*^6 months after study start vs study inclusion; ^**^12 months after study start vs study inclusion

### Laboratory analysis

A decrease of BNP levels was observed in eight patients after 6-month treatment of catechin. This decrease continued in seven patients 12 months after the treatment (Additional file 2: Figure S2). Mean levels of hsTnI, MYO and CKMB in the patients remained unchanged in the whole study. Results of cardiac markers and BNP are illustrated in Additional file 1: Table S4. Hepatic functions evaluated by ALT, AST, GGT and LDH, and renal functions presented by BUN, CREA and URCA remained unchanged in the patients before and after catechin administration (Additional file 1: Table S5).

### Genetic analysis

**Fig. 2:**
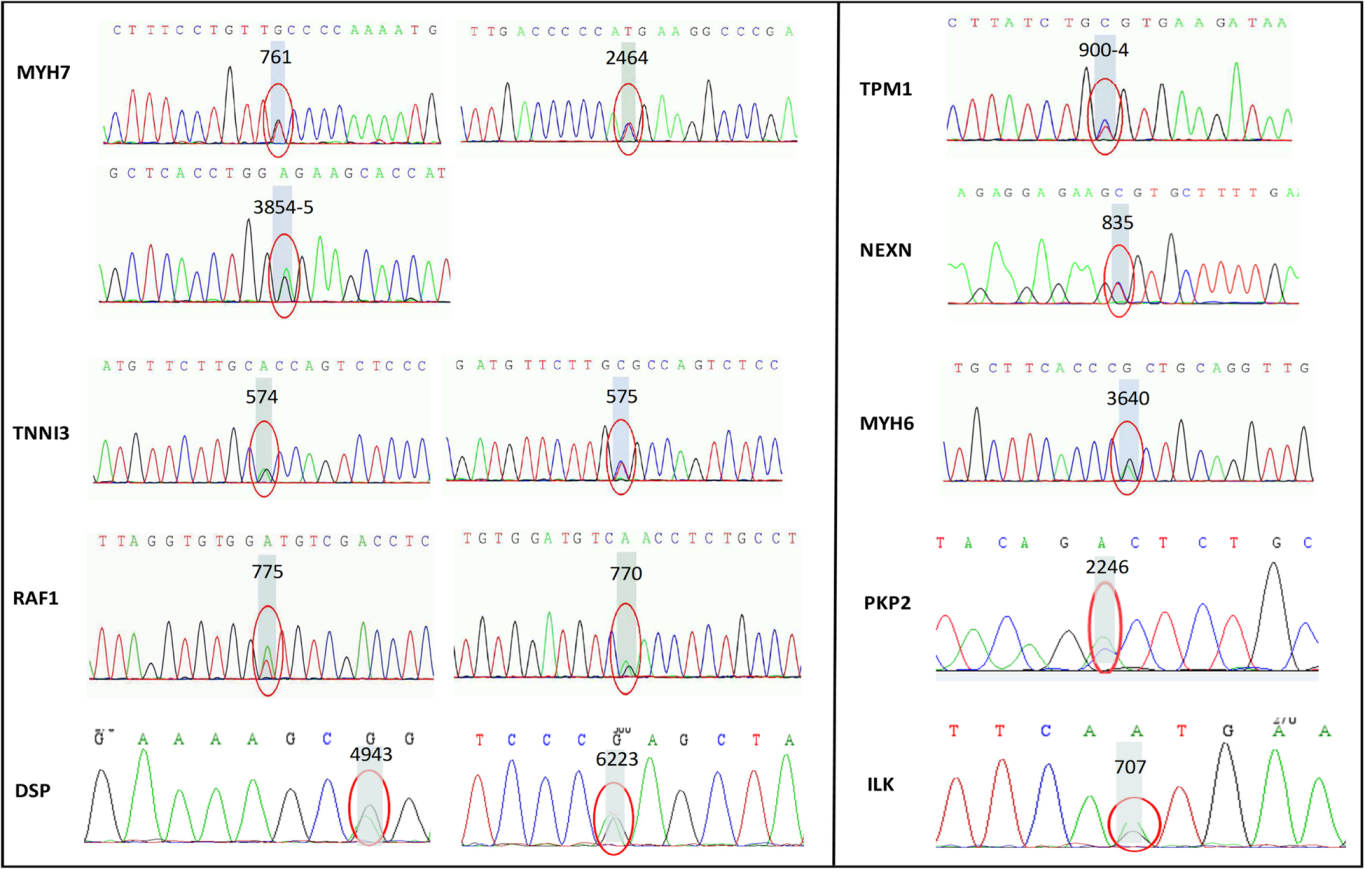
Sanger validation of the mutation sites in pediatric cardiomyopathy patients. *DSP*, desmoplakin; HCM, hypertrophic cardiomyopathy; HF, heart failure; *ILK*, integrin linked kinase; *MYH6*, alpha-myosin heavy chain; *MYH7*, beta-myosin heavy chain; NYHA, New York Heart Association; *NEXN*, nexilin F-actin binding protein; *PKP2*, plakophilin; *RAF1*, Raf-1 proto-oncogene, serine/threonine kinase

Genetic analysis of all patients is shown in Table 2 and Fig. 2. Of twelve patients, nine (58.3%) patients were carriers of gene variants inherited from parents or spontaneously. Of three dead patients, all carried gene mutations. Among them, two carried multigene mutations. Of nine survived patients, four patients had single-gene mutations and three patients had no definite variants. In total, 14 different variants were found, including six pathogenic variants (which have been reported associated with cardiomyopathy) and eight uncertain-significance variants. Among five HCM patients, the variant of beta-myosin heavy chain (*MYH7*) was detected in two HCM patients. However, the *MYH7* Ala254Glu (c.761C > A) mutation has not been reported so far in human. The other two HCM patients had multigene mutations and shared Raf-1 proto-oncogene, serine/threonine kinase (*RAF1*) gene variant (c.775 T > A, p.Ser259Thr; c.770C > T, p.Ser257Leu). In addition, the variants of alpha-myosin heavy chain (*MYH6*) and tropomyosin alpha-1 chain (*TPM1*) were also found in two HCM patients. The variant Arg279Cys (c.835C > T) of nexilin F-actin binding protein (*NEXN*) was detected in one HCM patient and this is a novel variant and not reported previously. Gene mutations were detected in four RCM patients as well. Variants Arg192Cys (c.574C > T) and Arg192His (c.575G > A) of isoform of troponin I (*TNNI3*) both leading to amino acid changes at the point of 192 were observed in two RCM patients, respectively. Additionally, the compound heterozygous mutations (p.Gln1648Arg, c.4943A > G and p.Arg2075Trp, c.6223C > T) of desmoplakin (*DSP*) inherited from his healthy parents was found in one RCM patient, which was extremely rare and never reported in RCM. The variants of integrin linked kinase (*ILK*)*,* plakophilin 2 (*PKP2*) and *MYH7* were also observed in RCM patients.

## Discussion

In this study, we have demonstrated the beneficial effect of daily administration of catechin in cardiomyopathy patients with diastolic dysfunction. Significant increases of LVEDV and SV were observed by echocardiography examination in the patients after 6-month treatment of catechin. In addition, an improvement of the E/A ratio was also detected in the patients after 12-month catechin administration. No significant changes in cardiac structure such as the wall thickness were observed in HCM patients nor atrial size changes in RCM patients during the study period. No significant changes of pulmonary arterial pressure were observed in all patients tested. No adverse side effects (significant ECG changes or abnormal functional changes) were observed in all patient treated with catechin during the whole study.

HCM, accounting for about 42% of pediatric cardiomyopathies [20], is described as a hypertrophic heart with increased cardiac wall thickness. For the moment, there is no effective therapy for the disease. In the past, most cardiomyopathy cases were described as idiopathic cardiomyopathies and the etiologies were unknown [21, 22]. However, recent genetic and molecular studies have demonstrated that most cardiomyopathy cases have genetic defects and some are inheritable [23]. So far, the common disease genes for HCM have been summarized as those of *MYH7*, *MYH6*, *TPM1* and *RAF1* [24–27]. In our study, we also identified that *MYH7* mutation is one of the most common gene variants in HCM cases. The two HCM patients with *MYH7* mutation shared a common echocardiographic feature i.e. increased interventricular septum and diastolic dysfunction, although their mutation points were different. *MYH6* mutation p.Arg1214Trp and *TPM1* splicing mutation in two HCM patients inherited from their healthy mothers indicate that the variants might not cause the disease. However, *RAF1* mutations p.Ser259Thr and p.Ser257Leu in these two HCM patients are pathogenic [28, 29], which was consistent with their clinical symptoms and signs. *NEXN* mutation p. Arg279Cys from the patient’s heathy father was very unlikely to cause the disease, and the etiology in this case need further study. RCM shares a common pathological manifestation as that of HCM, i.e. diastolic dysfunction. RCM is the least common type of cardiomyopathy, but with a high mortality, in pediatric cardiomyopathy cases. RCM is characterized by a biatrial enlargement and increased myocardial stiffness with a normal left ventricular internal dimension [22, 30]. Like HCM, most RCM cases have been described as idiopathic RCM in the previous studies, however, some sarcomere gene mutations have been reported that are associated with the development of RCM [31]. In this study, of seven RCM patients, myocardial gene mutations were detected in four patients. Among them, the *TNNI3* mutation leading acidic changes at the point of 192 was found in two patients confirming that *TNNI3* mutation is indeed a pathogenic cause for RCM [31–34]. In addition, one RCM patient with *TNNI3* mutation also carried a pathogenic *PKP2* mutation p.Ala749Asp [35], and her clinical manifestations were more serious than the patient who carries only single *TNNI3* mutation. *DSP* mutation, being associated with arrhythmogenic right ventricular cardiomyopathy, was detected as well in one RCM patient, which is never reported in RCM cases previously. Interestingly, this patient’s brother was also clinically diagnosed as RCM seven years ago (no genomic data) and died from heart failure one year later. The heterozygous mutations in *DSP*, inherited from his heathy parents, might be the cause of the disease, and the pathogenicity of the mutations need further study to confirm. Having combined the genotyping with phenotyping analyses in the patients of our study, we have found that patients with multigene mutations have a poorer outcome compared to those with single-gene mutations or without detectable gene mutations.

For the moment, most medications used for cardiomyopathy or diastolic dysfunction are anti-symptoms, such as diuretics and beta-blockers [6, 7, 36]. Very recently, however, several studies have demonstrated that desensitization could be useful in correcting Ca^2+^ hypersensitivities caused by myofibril protein mutations in various cardiomyopathies with diastolic dysfunction [8, 10, 11]. Catechin, the most abundant bioactive ingredient in green tea, has been confirmed to possess an ability of desensitizing myofilaments to Ca^2+^ by forming a ternary complex with the C-terminal domain of troponin C and the anchoring site of troponin I [11]. In vitro assays have revealed that acute treatment with 5 μM of catechin has a direct effect in myocardial cells isolated from RCM transgenic mice by reducing the affinity of TnC to Ca^2+^ and correcting impaired relaxation [8]. In RCM transgenic mice, a long-term treatment with catechin can improve diastolic function and reverse impaired relaxation [8]. The ability of catechin to diminish myofilament Ca^2+^ hypersensitivity has also been exhibited in a HCM mouse model, suggesting a therapeutic potential of this compound for treating diastolic dysfunction [10]. Other studies have shown that it can reduce left ventricular thickness and mass in patients after a daily intake of green tea over 12 months [37–39].

In the present study, the significant correction of clinical manifestations and cardiac dysfunction has been observed in the patients taking green tea extract catechin. Interestingly, RCM patients with a mutation of *TNNI3* have a better effect in cardiac function indicated by echocardiography measurements and BNP levels. However, due to the limited patient number, more studies are needed to confirm this hypothesis. Some studies have revealed that diastolic dysfunction can reduce cardiac output evaluated by stroke volume without much changes in EF in diastolic heart failure patients, i.e. heart failure with preserved ejection fraction (HFpEF) [40, 41]. We further confirm that the HCM or RCM patients with diastolic dysfunction in this study have a normal or nearly normal EF. BNP, another prognostic marker for cardiac function, is associated with cardiovascular mortality and hospitalization [42, 43]. In the present study, a reduction of BNP is observed in most of the patients tested.

Regarding the safety of catechin use, we think that green tea extract catechin is safe because it is one of the daily supplements recommended for use for many years without reports of any serious side effects [44]. In this study, we have examined and measured cardiac biomarkers, cardiac electrical activities, and hepatic and renal functional indications, all of which do not show any significant changes during the whole period of the study. Our observations are consistent with the reports from the other studies [37–39].

Due to limited sample sizes, double-blind, placebo-controlled trial could not be processed for further analysis. Based on this study, we are planning to carry on a randomised, multinational, double-blind trial to enroll more patients to confirm the therapeutical effects of catechin on diastolic dysfunction in cardiomyopathy patients.

## Conclusions

This study provides us with supporting evidences that green tea extract catechin can be used for cardiomyopathy patients with diastolic dysfunction due to its desensitizing effect. In addition, it is safe to use the green tea extract as a daily supplement under the doses used in this study without any obvious adverse side effects.

## Additional files

Additional file 1: **Table S1.** Cardiac function observed with electrocardiography in patients before and after the catechin administration. **Table S2.** Parameters measured with echocardiography in HCM patients. **Table S3.** Parameters measured with echocardiography in RCM patients. **Table S4.** Cardiac markers and BNP analysis. **Table S5.** Hepatic and renal functions. **Table S6.** NYHA classification. (DOCX 28 kb)

Additional file 2: **Figure S1.** Echocardiographic changes of cardiac parameters during the study period of 6 and 12 months. **Figure S2.** Levels of BNP during the study period. (DOCX 259 kb)

## Abbreviations

ACEI: Angiotensin-converting enzyme inhibitors
ALT: Alanine aminotransferase
AST: Aspertate aminotransferase
BNP: Brain natriuretic peptide
BUN: Urea nitrogen
CKMB: Creatine kinase isoenzyme
CREA: Creatinine
DCM: Dilated cardiomyopathy
DSP: Desmoplakin
EF: Ejection fraction
FS: Fractional shortening
GGT: Gamma-glutamyl transpeptidase
HCM: Hypertrophic cardiomyopathy
hsTnI: High-sensitivity troponin I
ILK: Integrin linked kinase
IVRT: Isovolumetric relaxation time
IVS: Interventricular septum
LDH: Lactate dehydrogenase
LV: Left ventricular
LVEDD: Left ventricle end diastolic dimension
LVEDV: Left ventricle end diastolic volume
LVESD: Left ventricle end systolic dimension
LVESV: Left ventricle end systolic volume
MYH6: Alpha-myosin heavy chain
MYH7: Beta-myosin heavy chain
MYO: Myoglobin
NEXN: Nexilin F-actin binding protein
NYHA: New York Heart Association
PAP: Pulmonary arterial pressure
PKP2: Plakophilin 2
RAF1: Raf-1 proto-oncogene, serine/threonine kinase
RCM: Restrictive cardiomyopathy
RVSP: Right ventricle systolic pressure
SV: Stroke volume
TNNI3: Isoform of troponin I
TPM1: Tropomyosin alpha-1 chain
URCA: Uric acid
