# Supplementary material for: Green tea extract catechin improves cardiac function in pediatric cardiomyopathy patients with diastolic dysfunction
Source: J Biomed Sci. 2019 May 8;26:32. doi: 10.1186/s12929-019-0528-7 (PMC6505250; doi:10.1186/s12929-019-0528-7)
Supplement: Supplementary file 1 — Table S1. Cardiac function observed with electrocardiography in patients before and after the catechin administration. Table S2. Parameters measured with echocardiography in HCM patients. Table S3. Parameters measured with echocardiography in RCM patients. Table S4. Cardiac markers and BNP analysis. Table S5. Hepatic and renal functions. Table S6. NYHA classification. (DOCX 28 kb) [file 12929_2019_528_MOESM1_ESM.docx]

Additional file 1

**Table S1. Cardiac function observed with electrocardiography in patients before and after the catechin administration**

| **Parameter** | **Study inclusion (n=12)** | **6 months after study start (n=10)** | ***P*-value^*^** | **12 months after study start (n=9)** | ***P*-value^**^** |
| --- | --- | --- | --- | --- | --- |
| HR (bpm) | 94±26 | 88±20 | ns | 86±22 | ns |
| P (ms) | 91±13 | 98±14 | ns | 95±28 | ns |
| P-R (ms) | 139±21 | 149±20 | ns | 146±33 | ns |
| QRS (ms) | 97±27 | 100±24 | ns | 99±22 | ns |
| QT (ms) | 361±58 | 361±46 | ns | 362±75 | ns |
| QTc (ms) | 428±36 | 431±34 | ns | 424±73 | ns |

**Notes:** HR, heart rate; ns, nonsignificance. Continuous data are expressed as mean±SD. ^*^6 months after study start vs study inclusion; ^**^12 months after study start vs study inclusion.

**Table S2. Parameters measured with echocardiography in HCM patients**

| **HCM** | **Study inclusion (n=5)** | **6 months after study start (n=4)** | ***P*-value^*^** | **12 months after study start (n=3)** | | ***P*-value^**^** |
| --- | --- | --- | --- | --- | --- | --- |
| IVS thickness (mm) | 16±3 | 19±6 | ns | 22±8 | ns | |
| LVPW thickness (mm) | 9±3 | 8±2 | ns | 10±6 | ns | |

**Notes:** IVS, intraventricular septum; LVPW, left ventricle posterior wall; ns, nonsignificance. Continuous data are expressed as mean±SD. ^*^6 months after study start vs study inclusion; ^**^12 months after study start vs study inclusion.

**Table S3. Parameters measured with echocardiography in RCM patients**

| **RCM** | **Study inclusion (n=7)** | **6 months after study start**  **(n=6)** | ***P*-value^*^** | **12 months after study start (n=6)** | ***P*-value^**^** |
| --- | --- | --- | --- | --- | --- |
| LA (mm) | 39±4 | 39±7 | ns | 40±8 | ns |
| RA (mm) | 42±7 | 45±7 | ns | 46±7 | ns |

**Notes:** LA, left atria; ns, nonsignificance; RA, right atria. Continuous data are expressed as mean±SD. ^*^6 months after study start vs study inclusion; ^**^12 months after study start vs study inclusion.

**Table S4. Cardiac markers and BNP analysis**

| **Parameter** | **Study inclusion (n=12)** | **6 months after study start (n=10)** | ***P*-value^*^** | **12 months after study start (n=9)** | | ***P*-value^**^** |
| --- | --- | --- | --- | --- | --- | --- |
| hsTnI (ng/ml) | 0.036±0.030 | 0.072±0.146 | ns | 0.045±0.035 | ns | |
| MYO (ng/ml) | 25.1±10.1 | 30.9±15.1 | ns | 32.1±13.7 | ns | |
| CKMB (ng/ml) | 3.9±3.2 | 4.4±2.9 | ns | 5.4±2.5 | ns | |
| BNP (pg/ml) | 2011.6±1566.6 | 958.6±605.5 | ns | 1034.6±864.7 | ns | |

**Notes:** BNP, brain natriuretic peptide; CKMB, creatine kinase isoenzyme; hsTnI, high-sensitivity troponin I; MYO, myoglobin; ns, nonsignificance.

Continuous data are expressed as mean±SD. ^*^6 months after study start vs study inclusion; ^**^12 months after study start vs study inclusion.

**Table S5. Hepatic and renal functions**

| **Parameter** | **Study inclusion (n=12)** | **6 months after study start (n=10)** | ***P*-value^*^** | **12 months after study start (n=9)** | ***P*-value^**^** |
| --- | --- | --- | --- | --- | --- |
| ALT (U/L) | 27.1±8.1 | 36.2±21.3 | ns | 31.9±12.4 | ns |
| AST (U/L) | 39.5±10.7 | 40.4±11.8 | ns | 42.4±11.2 | ns |
| GGT (U/L) | 59.8±47.5 | 92.8±78.7 | ns | 106.8±75.3 | ns |
| LDH (U/L) | 267.1±134.0 | 232.0±43.3 | ns | 242.6±58.8 | ns |
| BUN (mmol/L) | 5.1±1.3 | 5.7±1.5 | ns | 5.0±1.7 | ns |
| CREA (μmol/L) | 37.6±10.7 | 40.0±7.4 | ns | 39.3±10.4 | ns |
| URCA (μmol/L) | 372.4±118.2 | 413.4±115.4 | ns | 356.2±148.2 | ns |

**Notes:** ALT, alanine aminotransferase; AST, aspartate aminotransferase; BUN, blood urea nitrogen; CREA, creatinine; GGT, gamma-glutamyl transpeptidase; LDH, lactate dehydrogenase; URCA, uric acid; ns, nonsignificance. Continuous data are expressed as mean±SD. ^*^6 months after study start vs study inclusion; ^**^12 months after study start vs study inclusion.

**Table S6. NYHA classification**

| **Class** | **Description** |
| --- | --- |
| I | No limitation of physical activity. Ordinary physical activity does not cause symptoms of HF. |
| II | Slight limitation of physical activity. Comfortable at rest, but ordinary physical activity results in symptoms of HF. |
| III | Marked limitation of physical activity. Comfortable at rest, but less than ordinary activity causes symptoms |
| IV | Unable to carry on any physical activity without symptoms of HF, or symptoms of HF at rest. |

**Notes:** HF, heart failure; NYHA, New York Heart Association.
