# Supplementary material for: Green tea extract catechin improves cardiac function in pediatric cardiomyopathy patients with diastolic dysfunction
Source: J Biomed Sci. 2019 May 8;26:32. doi: 10.1186/s12929-019-0528-7 (PMC6505250; doi:10.1186/s12929-019-0528-7)

**Supplemental Figure Legends**

**Additional file 2: Figure S1. Echocardiographic changes of cardiac parameters during the study period of 6 and 12 months.**

**Notes:** (a) IVS thickness; (b) LVPW thickness; (c) LA; (d) RA detected by echocardiography in patients before (t_0_), after 6 (t_6_) and 12 months (t_12_) of green tea extract catechin. IVS, interventricular septum; LA, left atria; LVPW, left ventricle posterior wall; RA, right atria; t_0_, before the administration of catechin; t_6_, 6 months after the administration of catechin; t_12_, 12 months after administration of catechin.

**Figure S2. Levels of BNP during the study period.**

**Notes:** BNP, B type natriuretic peptide; t_0_, before the administration of catechin; t_6_, 6 months after the administration of catechin; t_12_, 12 months after administration of catechin.

Supplemental Figure S1
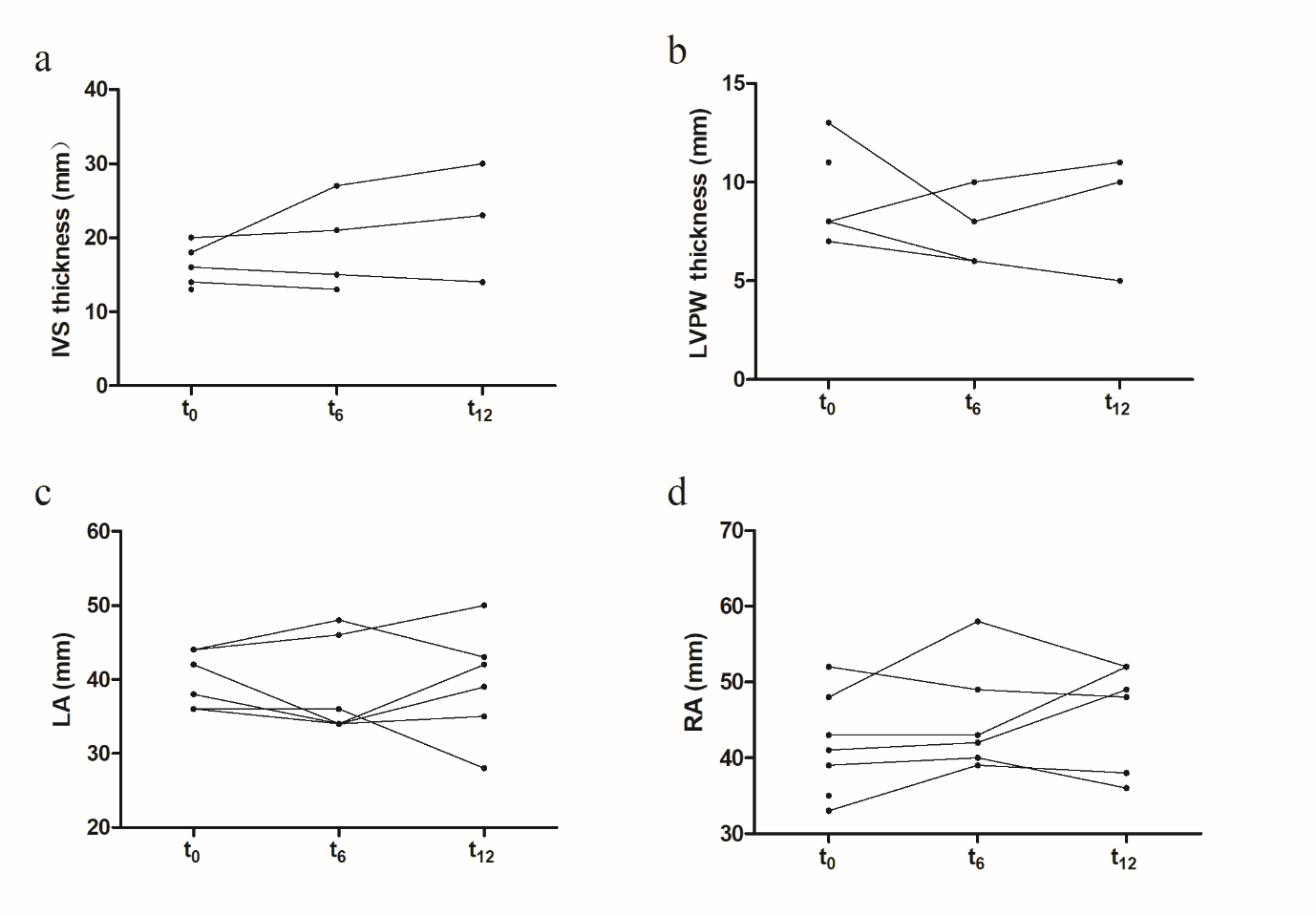


Supplemental Figure S2
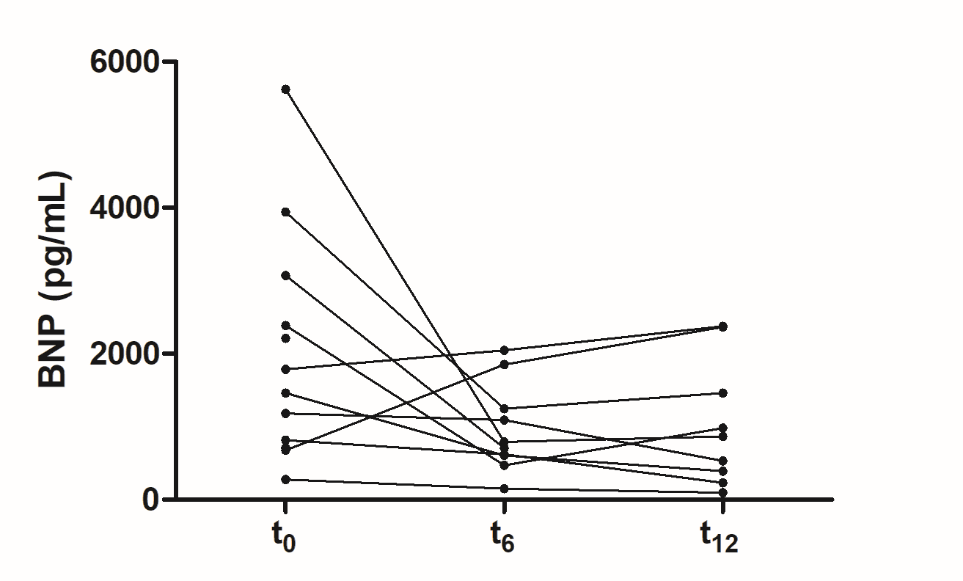

Supplement: Supplementary file 2 — Figure S1. Echocardiographic changes of cardiac parameters during the study period of 6 and 12 months. Figure S2. Levels of BNP during the study period. (DOCX 259 kb) [file 12929_2019_528_MOESM2_ESM.docx]
